# Thrombocytosis in patients with spondyloarthritis: a case–control study

**DOI:** 10.1186/s12891-023-06304-1

**Published:** 2023-03-15

**Authors:** Linan Deng, Pingping Zheng

**Affiliations:** 1 Department of Rheumatology, Fuzhou Second Hospital, 47 Shangteng Road, Cangshan District, Fuzhou, 350007 China; 2 Department of Burns and Wounds, Fujian Medical University Union Hospital, Fuzhou, China

**Keywords:** Spondyloarthritis, Thrombocytosis, White blood cells, Fibrinogen, Anti-TNF-α therapy

## Abstract

**Objective:**

This study aimed to investigate the clinical and laboratory as well as radiological features of spondyloarthritis (SpA) patients with thrombocytosis and to explore risk factor for thrombocytosis in SpA patients and to assess the effect of antitumor necrosis factor-α (anti-TNF-α) therapy on platelet count in SpA patients with thrombocytosis.

**Methods:**

A total of 145 patients with SpA were included in this study, and non-thrombocytosis was identified in 76 patients while thrombocytosis was found in 69 patients, 38 out of the 69 patients received anti-TNF-α therapy. Logistic regression analysis was performed to investigate risk factors that associated with thrombocytosis. The platelet count of patients in the thrombocytosis group treated with anti-TNF-α therapy on week 0, week 6 and week 12 were collected and compared with conventional therapy group.

**Results:**

The proportion of hip involvement (60.86% vs 36.84%, *p* = 0.004), bath ankylosing spondylitis disease activity index score (4.24 ± 0.55 vs 3.69 ± 0.67, *p* < 0.001), erythrocyte sedimentation rate (62.22 ± 41.97 mm/hour vs 27.00 ± 25.93 mm/hour, *p* < 0.001), C-reactive protein (53.45 ± 47.45 mg/L vs 18.91 ± 31.09 mg/L, *p* < 0.001), fibrinogen (5.77 ± 1.48 g/L vs 4.01 ± 1.32 g/L, *P* < 0.001), white blood cells (8.15 ± 1.90 × 10^9^/L vs 6.85 ± 2.39 × 10^9^/L, *p* < 0.001) and neutrophils (5.08 ± 1.55 × 10^9^/L vs 4.01 ± 2.04 × 10^9^/L, *p* = 0.001) are higher in thrombocytosis group, but hemoglobin and albumin are lower compared to non-thrombocytosis group (122.88 ± 17.25 g/L vs 131.51 ± 16.03 g/L, *p* = 0.002; 37.19 ± 4.73 g/L vs 39.67 ± 3.99 g/L, *p* = 0.001, respectively). Multivariable logistic regression analysis indicated that higher white blood cells (OR, 1.644; 95% CI, 1.045–2.587; *P* = 0.032) and fibrinogen (OR, 2.169; 95% CI, 1.237–3.804; *P* = 0.007) were independently associated with thrombocytosis in SpA patients. The platelet count in the thrombocytosis group treated with anti-TNF-α therapy on week 6 and week 12 were statistically lower than week 0 (225.05 ± 60.58 × 10^9^/L vs 368.26 ± 54.34 × 10^9^/L, *p* < 0.001; 201.26 ± 51.48 × 10^9^/L vs 368.26 ± 54.34 × 10^9^/L, *p* < 0.001) and conventional therapy (week 6, 225.05 ± 60.58 × 10^9^/L vs 370.00 ± 74.05 × 10^9^/L, *p* < 0.001; week 12, 201.26 ± 51.48 × 10^9^/L vs 303.13 ± 71.49 × 10^9^/L, *p* < 0.001).

**Conclusion:**

SpA patients with thrombocytosis have a higher proportion of hip involvement and disease activity compared to non-thrombocytosis SpA patients. The potential risk factors for thrombocytosis in SPA patients were higher white blood cells and fibrinogen. Anti-TNF-α therapy can reduce the increased platelets more effectively and rapidly than conventional treatments in SpA patients with thrombocytosis.

## Introduction

Spondyloarthritis (SpA) encompasses a heterogeneous group of inflammatory arthritis which share many clinical features and a genetic correlation with human leukocyte antigen B27 (HLA-B27) [1]. Typical clinical manifestation of SpA-related diseases ranges from axial symptom which is inflammatory back pain to peripheral presentations, such as arthritis, enthesitis and dactylitis. Extra-articular manifestations, such as uveitis, inflammatory bowel diseases and psoriasis sometimes can be observed in patients with SpA [2]. According to criteria proposed by the Assessment of Spondyloarthritis International Society (ASAS), patients with SpA can be classified into axial SpA (ax-SpA) and peripheral SpA (p-SpA) based on their different clinical manifestation [3, 4]. The term ax-SpA covers two subgroups: patients with definite radiographic changes of sacroiliac joint (SIJ) defined by modified New York criteria (radiographic axial spondyloarthritis[r-as-SpA], also labeled as ankylosing spondylitis [AS]), and patients without those changes on conventional radiographs (non-radiographic axial spondyloarthritis [nr-as-SpA]) [5]. The latter is regarded as an early stage of r-as-SpA, and inflammation in the SIJ can be detected by magnetic resonance imaging (MRI) before the definite radiographic change develops [6].

Platelets are anucleate fragments generated by megakaryocytes in bone marrow. Despite their well-established role in hemostasis and thrombosis, growing evidence indicates that platelets also play an integral role in innate and adaptive immunity [7, 8]. Previous studies have demonstrated that platelets are associated with the pathogenesis of multiple autoimmune diseases such as rheumatoid arthritis, systemic lupus and multiple sclerosis [7–10]. In SpA, numerous studies have revealed that platelet counts in patients with SpA are significantly higher than healthy controls, and the elevated platelet count were account for the increased cardiovascular mortality and morbidity in SpA [11–15]. Additionally, platelets were also reported to be related to the new bone formation, severity of inflammation and treatment response in SpA [16, 17], and many platelet-derived immune mediators such as platelet-derived growth factor, transforming growth factor beta were also found to be overexpressed in patients with SpA [18]. However, none of these studies analysis the characteristics of SpA patients with thrombocytosis and investigate risk factors that associated with thrombocytosis in SpA. Herein, we conducted a retrospective study to evaluate the clinical and laboratory and radiological features of SpA patients with thrombocytosis and to explore risk factors that associated with thrombocytosis and to assess the effect of antitumor necrosis factor-α (anti-TNF-α) therapy on platelet count in SpA patients with thrombocytosis.

## Methods

### Patients

A retrospective study was performed on 145 inpatients with SpA in Fujian Medical university union hospital during the period January 2017 to June 2022. The inclusion criteria were: (1) patients fulfilling the ASAS criteria for axial SpA or the ASAS criteria for peripheral SpA [3, 4]; (2) had a follow-up duration of more than 3 months. The exclusion criteria were as follows: (1) diagnosed with other autoimmune diseases; (2) treatment with anti-platelet drugs; (3) post splenectomy operation; (4) diagnosed with hematological diseases affecting the platelet. This study was approved by the ethics committee of Fujian Medical university union hospital, and informed consent was waived due to the retrospective nature of the study design.

## Date collection

Demographic data (gender, age at diagnosis and family history of SpA), initial symptoms (axial, peripheral and extra-articular symptoms), ASAS classification and laboratory parameters (platelet count, hemoglobin, red blood cells, white blood cells, neutrophils, lymphocyte, monocyte, erythrocyte sedimentation rate, C-reactive protein, D-dimer, fibrinogen, albumin and the result of HLA-B27 at first admission) were documented from electronic medical records. Imaging finding, including MRI scan, computer tomography (CT) scan and plane X-rays of the related joints such as SIJ, hip joint and spine were collected. Sacroiliitis on MRI was defined according to the ASAS criteria, as follows: (1) Bone marrow edema (BMO) on a T2-weighted sequence or bone marrow contrast enhancement on a T1-weighted sequence has to be clearly present and located in a typical anatomical area such as subchondral bone. (2) MRI appearance must be highly suggestive of SpA. Definite radiographic sacroiliitis was defined by modified New York criteria (grade 3–4 unilateral or grade 2 bilateral) [19]. Hip joint involvement was defined as hip pain or limited mobility of hip/hips or both acute and chronic inflammatory changes on MRI or radiographic hip joint involvement defined by The Bath Ankylosing Spondylitis Index (BASRI-hip) score > 2 [20]. Bath Ankylosing Spondylitis Disease Activity Index (BASDAI) [21] score was obtained from electronic medical records to evaluate disease activity, and a cut off value score of 4 was used to discriminate SpA patients of active group from remission group. The normal reference level of platelet count was 100–300 × 10^9^/L according to the laboratory criteria of Fujian Medical university union hospital. Therefore, thrombocytosis is considered a platelet count exceeding 300 × 10^9^/L, and non-thrombocytosis was defined as a blood platelet ≤ 300 × 10^9^/L.

## Statistical analysis

Continuous data were presented as mean ± standard deviation and examined by Student’s t-test or paired t-test or one-way ANOVA. Categorical data were expressed as proportion and compared using the Chi-square or Fisher’s exact test. Univariable and multivariable logistic regression analysis was used to identify factors that associated with thrombocytosis in patients with SpA, and variables with *P*-value < 0.1 in univariable regression analysis was included in multivariable logistic regression analysis. All statistical analysis was performed by SPSS version 26.0 (IBM) and GraphPad Prism 8.0, and a two-side *P*-value < 0.05 indicated statistically significant.

## Results

### Comparisons between SpA patients with thrombocytosis and non-thrombocytosis

A total of 145 patients with SpA were included in this study, and thrombocytosis was found in 69 patients (47.59%), and non-thrombocytosis was identified in 76 patients (52.41%). The platelet counts of non-thrombocytosis group were significantly lower than thrombocytosis group (239.51 ± 41.08 × 10^9^/L vs 373.64 ± 63.70 × 10^9^/L, *p* < 0.001). Compared to SpA patients with non-thrombocytosis, those with thrombocytosis had a higher proportion of hip involvement (60.86% vs 36.84%, *p* = 0.004) and BASDAI score (4.24 ± 0.55 vs 3.69 ± 0.67, *p* < 0.001). Erythrocyte sedimentation rate (62.22 ± 41.97 mm/hour vs 27.00 ± 25.93 mm/hour, *p* < 0.001), C-reactive protein (53.45 ± 47.45 mg/L vs 18.91 ± 31.09 mg/L, *p* < 0.001), fibrinogen (5.77 ± 1.48 g/L vs 4.01 ± 1.32 g/L, *P* < 0.001), white blood cells (8.15 ± 1.90 × 10^9^/L vs 6.85 ± 2.39 × 10^9^/L, *p* < 0.001) and neutrophils (5.08 ± 1.55 × 10^9^/L vs 4.01 ± 2.04 × 10^9^/L, *p* = 0.001) in thrombocytosis group were higher than non-thrombocytosis group. Albumin (37.19 ± 4.73 g/L vs 39.67 ± 3.99 g/L, *p* = 0.001) and hemoglobin (122.88 ± 17.25 g/L vs 131.51 ± 16.03 g/L, *p* = 0.002) were lower in thrombocytosis group compared with non-thrombocytosis group. No statistically significant difference was observed between the two groups in terms of gender, age, family history of SpA, clinical manifestations, ASAS classification, HLA-B27 results, monocytes, lymphocytes, red blood cells, D-dimer, sacroiliitis on MRI and radiographic sacroiliitis (Table 1).

**Table 1:**
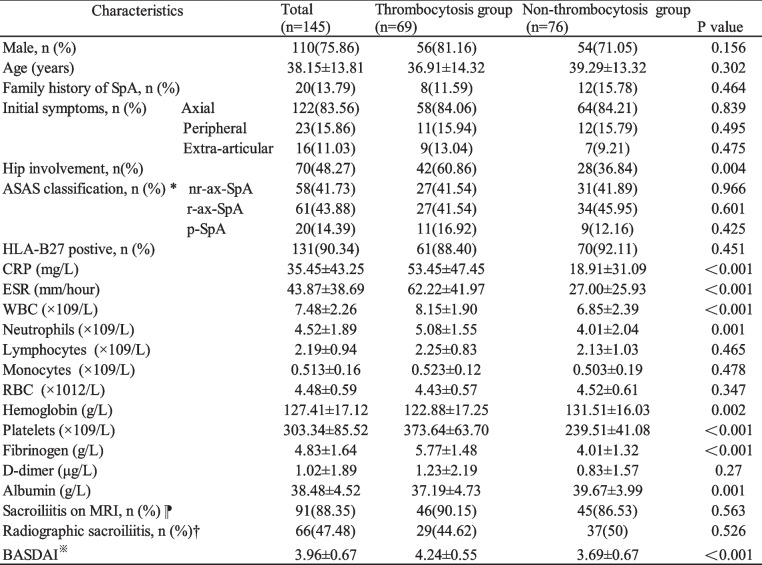
Comparison of demographic features and laboratory parameters between spondyloarthritis patients with thrombocytosis and non-thrombocytosis

*Available in 139 patients (*n* = 65 for the thrombocytosis group and *n* = 74 for the non-thrombocytosis group)

⁋ Available in 103 patients (*n* = 51 for the thrombocytosis group and *n* = 52 for the non-thrombocytosis group)

† Available in 139 patients (*n* = 65 for the thrombocytosis group and *n* = 74 for the non-thrombocytosis group)

^※^Available in 125 patients (*n* = 60 for the thrombocytosis group and *n* = 65 for the non-thrombocytosis group)

*SpA* spondyloarthritis, *ASAS* Assessment of Spondyloarthritis International Society, *nr-ax-SpA* non-radiographic axial spondyloarthritis, *r-ax-SpA* radiographic axial spondyloarthritis, *p-SpA* peripheral spondyloarthritis, *HLA-B27* human leukocyte antigen B27, *WBC* white blood cells, *RBC* red blood cells, *CRP* C-reactive protein, *ESR* erythrocyte sedimentation rate, *MRI* magnetic resonance imaging, *BASDAI* Bath Ankylosing Spondylitis Disease Activity Index

## Risk factors of thrombocytosis in patients with SpA

Logistic regression analysis indicated that higher proportion of hip involvement (OR, 2.667; 95% CI, 1.362–5.219; *P* = 0.004), higher BASDAI score (OR, 3.839; 95% CI, 2.070–7.122; *P* < 0.001), higher erythrocyte sedimentation rate (OR, 1.031; 95% CI, 1.018–1.044; *P* < 0.001), higher C-reactive protein (OR, 1.024; 95% CI, 1.013–1.035; *P* < 0.001), higher fibrinogen (OR, 2.373; 95% CI, 1.747–3.223; *P* < 0.001), higher white blood cells (OR, 1.334; 95% CI, 1.123–1.585; *P* < 0.001), higher neutrophils (OR, 1.423; 95% CI, 1.144–1.769; *P* = 0.002), lower albumin (OR, 0.878; 95% CI, 0.810–0.951; *P* = 0.001) and hemoglobin (OR, 0.969; 95% CI, 0.950–0.989; *P* = 0.003) were statistically significant associated with thrombocytosis in patients with SpA. Variables identified in the univariate logistic regression analysis were included in the multivariable logistic regression analysis, and the results revealed that higher fibrinogen (OR, 2.169; 95% CI, 1.237–3.804; *P* = 0.007) and white blood cells (OR, 1.644; 95% CI, 1.045–2.587; *P* = 0.032) were independently associated with thrombocytosis in patients with SpA (Table 2).

**Table 2:**
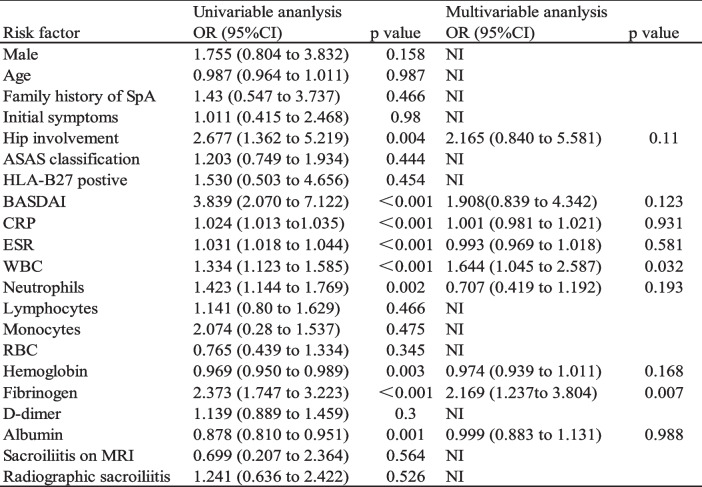
Risk factors for thrombocytosis in patients with spondyloarthritis in univariable and multivariable analysis

*SpA* spondyloarthritis, *ASAS* Assessment of Spondyloarthritis International Society, *HLA-B27* human leukocyte antigen B27, *BASDAI* Bath Ankylosing Spondylitis Disease Activity Index, *CRP* C-reactive protein, *ESR* erythrocyte sedimentation rate, *WBC* white blood cells, *RBC* red blood cells, *MRI* magnetic resonance imaging. *NI* not included in the multivariate analysis

## The effect of antitumor necrosis factor-α therapy on platelet count in SpA patients with thrombocytosis

**Fig. 1:**
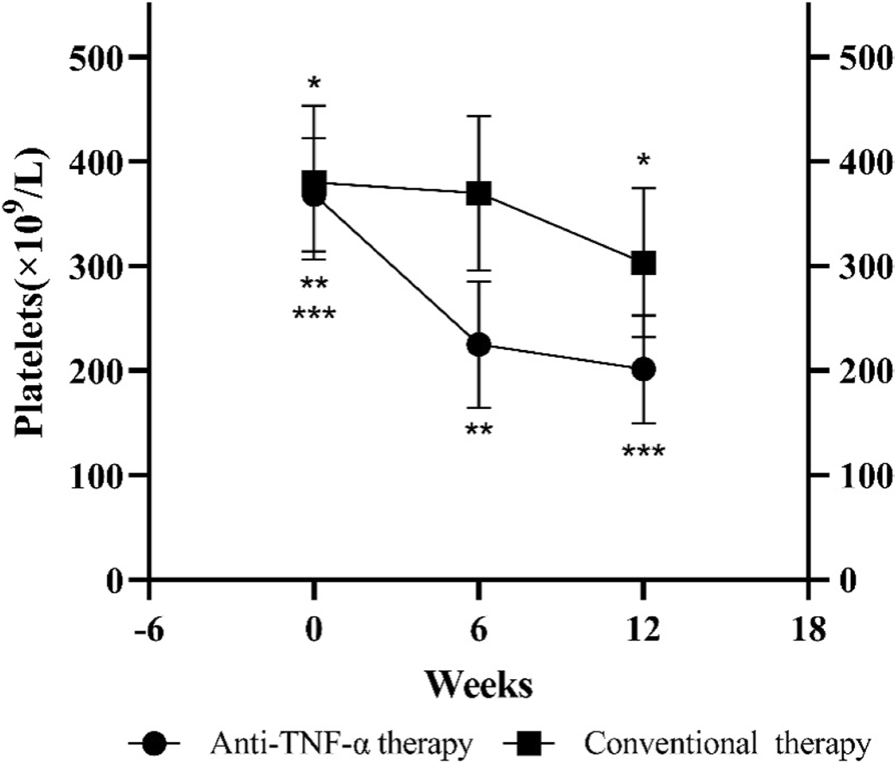
Platelet count in spondyloarthritis patients with thrombocytosis treated with anti-TNF-α therapy and conventional therapy. * *p* < 0.05 for 31 spondyloarthritis patients with thrombocytosis treated with conventional therapy between week 12 and week 0. ** *p* < 0.05 for 38 spondyloarthritis patients with thrombocytosis treated with anti-TNF-α therapy between week 6 and week 0. *** *p* < 0.05 for 38 spondyloarthritis patients with thrombocytosis treated with anti-TNF-α therapy between week 12 and week 0

A total of 31 patients in the thrombocytosis group were treated with conventional therapy (nonsteroidal anti-inflammatory drug and disease-modifying antirheumatic drug only). 38 patients in the thrombocytosis group were treated with anti-TNF-α therapy, and among them 35 patients were treated with etanercept, 2 patients were treated with adalimumab and 1 patient was treated with infliximab. The baseline platelet count (week 0) of anti-TNF-α therapy group and conventional therapy group were 368.26 ± 54.34 × 10^9^/L and 380.23 ± 73.99 × 10^9^/L, respectively (Table 3), and no statistically significant difference was observed between the two groups (*p* = 0.442). After 6- and 12-weeks treatment, the platelet count of the anti-TNF-α therapy group were statistically lower than conventional therapy group (225.05 ± 60.58 × 10^9^/L vs 370.00 ± 74.05 × 10^9^/L, *p* < 0.001; 201.26 ± 51.48 × 10^9^/L vs 303.13 ± 71.49 × 10^9^/L, *p* < 0.001). For the anti-TNF-α therapy group, the platelet count of week 6 and week 12 were statistically lower than week 0 (225.05 ± 60.58 × 10^9^/L vs 368.26 ± 54.34 × 10^9^/L, *p* < 0.001; 201.26 ± 51.48 × 10^9^/L vs 368.26 ± 54.34 × 10^9^/L, *p* < 0.001), and for conventional therapy group, the platelet count of week 12 were statistically lower than week 0 (303.13 ± 71.49 × 10^9^/L vs 380.23 ± 73.99 × 10^9^/L, *p* < 0.001), and no statistically significant difference was detected between the week 6 and week 0 (370.00 ± 74.05 × 10^9^/L vs 368.26 ± 54.34 × 10^9^/L, *p* = 0.078) of the platelet count (Fig. 1).

**Table 3:**
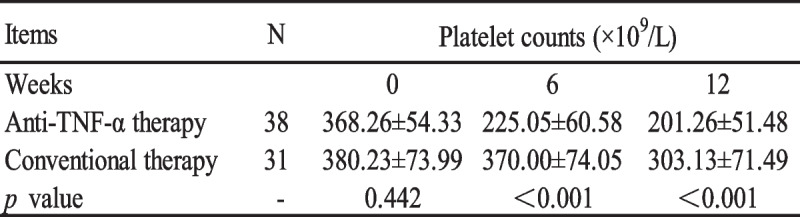
Comparison of platelet count between spondyloarthritis patients with thrombocytosis treated with anti-TNF-α therapy and conventional therapy

## Discussion

The first part of the study came out that SpA patients with thrombocytosis have a higher proportion of hip involvement, BASDAI score, erythrocyte sedimentation rate, C-reactive protein, fibrinogen, white blood cells and neutrophils, but hemoglobin and albumin are lower compared to non-thrombocytosis group.

SpA is an autoinflammatory disease and inflammation was associated with the pathogenesis of SpA [2]. Erythrocyte sedimentation rate and C-reactive protein are well-established inflammatory indicators that elevated in SpA patients with active disease [11]. Besides, numerous studies reported that a lower albumin and a higher fibrinogen, neutrophils and BASDAI score were observed in SpA patients with more severe disease [13, 22–25]. These studies validated that fibrinogen, neutrophils and albumin could act as novel biomarkers to reflect disease activity in SpA. Our data show that BASDAI score, erythrocyte sedimentation rate, C-reactive protein, fibrinogen, white blood cells and neutrophils are higher while albumin is lower in SpA patients with thrombocytosis compared to patients with non-thrombocytosis, indicating that SpA patients with thrombocytosis have a high disease activity. Interesting, this study found that a lower hemoglobin was observed in SpA patients with thrombocytosis. Hemoglobin is an important parameter to reflect nutrition status. Anemia is a common complication of inflammatory disease, and some studies reported that 6%-25% of the SpA patients have anemia [26, 27]. In rheumatoid arthritis, anemia is proven to be associated with disease severity [28]. A study found that hemoglobin was lower in AS patients with active disease group than those with stable disease group [27]. Altogether, these data suggest that the disease is more severe in SpA patients with thrombocytosis.

Hip involvement frequently occurs in patients with SpA. Our data show that 60.86% of SpA patients with thrombocytosis have hip involvement, which was slightly higher than previous studies that reported that hip involvement was observed in10-50% of the SpA patients [20]. In SpA patients with non-thrombocytosis group, the proportion of hip involvement was 38.15%, which was lower than thrombocytosis group. One explanation of this is that hip involvement was reported as a classical feature in patients with more severe disease activity [20, 29, 30], and in our study the disease is more severe in thrombocytosis group. Besides, in patients with osteoarthritis, hip osteoarthritis severity was found associate with platelet count. An observational study revealed that a higher platelet count was observed in severe hip osteoarthritis than in mild-moderate hip osteoarthritis (259.18 ± 71.39 × 109/L vs 239.66 ± 33.30 × 109/L, *p* = 0.03) [31], and a Korean study show that an elevated platelet count is associated with the presence of radiographic hip osteoarthritis in woman [32]. Our study also found that hip involvement was associated with thrombocytosis in SpA in the univariate logistic regression analysis. All these findings suggest that hip involvement have a correlation with platelet counts.

We then explore risk factors associated with thrombocytosis in SpA patients, and our data indicated that higher white blood cells and fibrinogen were associated with thrombocytosis.

Animal model and studies of human tissue demonstrated that white blood cells can bind to activated platelet, and platelet recruitment of white blood cells has been approved to be associated with many inflammatory processes in animal model [33, 34]. Accumulating evidence shows that platelet and white blood cells including neutrophils, lymphocytes and monocytes play crucial roles in the inflammatory process of SpA [12, 35, 36]. Early studies reported that white blood cells and it subtype counts such as neutrophils to lymphocytes ratio, platelet to lymphocytes ratio and monocyte to lymphocytes ratio were higher in patients with SpA compare to health controls, and these parameters could act as novel inflammatory indicators to reflect disease activity in SpA [11, 12, 37]. Consistently, our data indicated that a higher white blood cells was observed in thrombocytosis group which have a more severe disease activity, and both univariate and multivariable logistic regression analysis revealed that white blood cells were independently associated with thrombocytosis in patients with SpA.

Fibrinogen was also considered as an acute phase response protein and was reported that could reflect disease activity in patients with SpA [23, 38]. Recently, two studies show that fibrinogen was statistically higher in SpA patients than healthy controls, and subgroup analysis indicated that platelet counts and fibrinogen were both elevated in SpA patients with a BASDAI score ≥ 4 [13, 23]. Besides, fibrinogen and platelet were key elements of coagulation and fibrinolytic system, and the interactions between fibrinogen and platelet are involved in hemostasis and thrombosis [39]. Previous studies indicated that elevated fibrinogen level and platelet counts are two factors that associated with an increased cardiovascular mortality and morbidity in patients with SpA [14]. Taken together, all these studies underscore a relationship between fibrinogen and platelet in SpA. Consistent with these findings, our study revealed that higher fibrinogen was one of the factors which associated with thrombocytosis in SpA.

The last part of the study demonstrated that anti-TNF-α therapy can reduce the increased platelets more effectively and rapidly than conventional therapy in SpA patients with thrombocytosis.

Anti-TNF-α therapy can be classified in five groups based on different drugs: infliximab, adalimumab, etanercept, golimumab and certolizumab pegol. Many studies and meta-analysis found that platelet count can be reduced by anti-TNF-α therapy in SpA patients and other autoimmune diseases such as rheumatoid arthritis and psoriasis [17, 40, 41]. One study found that the increased platelet counts in AS patients were significantly reduced after 6 months of anti-TNF-α therapy (309 ± 70.00 × 109/L vs 342 ± 69.00 × 109/L) [40]. Consistent with these findings, our data indicated that the platelet count in the thrombocytosis group was statistically lower than baseline platelet count after 6 weeks and 12 weeks anti-TNF-α therapy. Additionally, our study firstly compared the anti-TNF-α therapy with convention therapy in terms of the platelet-lowing effect. In line with early findings [40], our data suggested that convention therapy can also reduce the increase platelet count. But unlike the anti-TNF-α therapy, the platelet count was statistically lower only after 12 weeks of convention treatment, indicating that anti-TNF-α therapy can reduce the increased platelets more effectively and rapidly than conventional treatments. Though the exact mechanism of the platelet-lowing effect of anti-TNF-α therapy remains unclear, TNF-α and its receptor were believed to be associated with this phenomenon. Previous works have confirmed that TNF receptors are expressed by thrombocytes and that TNF-α contributes to platelet-biased hematopoiesis and the hyperreactivity of platelets in bone marrow [17, 42, 43], indicating that anti-TNF-α therapy may have a direct inhibitory effect on platelet production by affecting platelet-biased hematopoiesis. Altogether, our date indicated that anti-TNF-α therapy can effectively reduce increased platelet counts in SpA patients with thrombocytosis.

This study has some limitations. First, it was a single-center study with a relatively small sample size, so the findings of our study need further validation by multicenter study with larger samples. Second, selection bias and recall bias were unavoidable due to the retrospective nature of the study design. Third, some data were incomplete or missing, thus other important parameters used in the evaluation disease activity of SpA such as ASDAS-ESR, ASDAS-CRP were not analysed in the study.

## Conclusions

In summary, SpA patients with thrombocytosis have a higher proportion of hip involvement and disease activity compared to non-thrombocytosis group. The potential risk factors for thrombocytosis in SPA patients were higher white blood cells and fibrinogen. Anti-TNF-α therapy can reduce the increased platelets more effectively and rapidly than conventional therapy in SpA patients with thrombocytosis.

## Data Availability

The datasets used during the study are available from the corresponding author upon reasonable request.

## Abbreviations

SpA: Spondyloarthritis
anti-TNF-α: Antitumor necrosis factor-α
HLA-B27: Human leukocyte antigen B27
ASAS: The Assessment of Spondyloarthritis International Society
ax-SpA: Axial spondyloarthritis
p-SpA: Peripheral spondyloarthritis
SIJ: Sacroiliac joint
r-as-SpA: Radiographic axial spondyloarthritis
AS: Ankylosing spondylitis
nr-as-SpA: Non-radiographic axial spondyloarthritis
MRI: Magnetic resonance imaging
CT: Computer tomography
BASRI-hip: The Bath Ankylosing Spondylitis Index
BASDAI: Bath Ankylosing Spondylitis Disease Activity Index
